# Vitamin D Supplementation in Australia: Implications for the Development of Supplementation Guidelines

**DOI:** 10.1155/2014/374208

**Published:** 2014-08-19

**Authors:** Kellie Bilinski, Peter Talbot

**Affiliations:** ^1^Westmead Breast Cancer Institute, Westmead Hospital and The University of Sydney, P.O. Box 143, Westmead, NSW 2145, Australia; ^2^Department of Dietetics & Nutrition, Westmead Hospital, Westmead, NSW 2145, Australia

## Abstract

High rates of vitamin D deficiency and testing have been reported in Australia, yet there are few reports regarding vitamin D supplement use. Australian wholesale sales data was obtained for vitamin D supplements for the period 2000–2011. There has been a threefold increase in supplement sales over the past decade, whereby over A$94 million supplements containing vitamin D in Australia were sold during the year 2010. There were eighty-nine manufacturers that produce a variety of 195 vitamin D products. The amount of vitamin D in these products varies considerably, from 40 to 1000 IU per unit, although supplements containing only vitamin D had the highest amount of vitamin D. There was a trend for sales to increase in winter months. Given the potential public health benefits of vitamin D, there is an urgent need for a better understanding of supplementation use and for the development of supplementation.

## 1. Introduction 

A high prevalence of vitamin D deficiency (25-hydroxyvitamin D (25(OH)D) ≤50 nmol/L) in Australians has been reported [1]. The Australian Health Survey showed that one-quarter (23.5%) of Australians are vitamin D deficient, 17% of whom had mild deficiency (20–40 nmol/L); 6% had moderate deficiency (13–29 nmol/L), and <1% had severe deficiency (<13 nmol/L). Serum vitamin D levels varied by season whereby deficiency rates ranged from 36% in winter to 14% in summer. The highest prevalence of vitamin D deficiency occurred in the south eastern states of Victoria (49%), ACT (49%), and Tasmania (43%), whereas, in the northern states of Queensland and the Northern Territory, the prevalence of vitamin D deficiency was 15% and 17%, respectively.

This may be driving the 95-fold increase in testing of 25(OH)D concentration in Australia reported over the last decade [2]. In addition to osteoporosis, achieving adequate vitamin D has been linked with protection from numerous chronic diseases including diabetes and cancer [3]. Exposure of the skin to ultraviolet-B (UVB) radiation from sunlight provides the body's principal source of vitamin D [4], although it can also be obtained by diet and supplements.

It has been assumed that most individuals achieve adequate sunlight exposure to meet their requirements [5]. However, adherence to sun protection messages [6] dramatically reduces 25(OH)D synthesis. We have previously shown that there are few opportunities in Australia to obtain the equivalent of 1000 IU 25(OH)D within a realistic duration while adhering to sun smart messages [7].

It is difficult to meet vitamin D requirements from the diet as few foods contain adequate amounts of vitamin D [8]. It has been estimated that Australians consume approximately 80 and 176 IU vitamin D from foods and supplements daily [9, 10]. This is despite the mandatory fortification of vitamin D in Australia of edible oils and spreads, whereby these foods must contain a minimum of 220 IU vitamin D per 100 g of food and voluntary fortification of modified and skim milk and powdered milk, yoghurts, and table confections (as well as cheese) [11]. This estimated intake is considerably lower than the estimated average requirements publicized by the US Institute of Medicine (IOM) of 400 IU/d for all adults [12] and the current adequate intake (AI) values for Australia of 200 IU for adults up to age of 50 years, 400 IU for those aged 50 to 70 years, and 600 IU for those aged 70 years [13].

Supplementation has been suggested as a safe and effective way of meeting 25(OH)D requirements [14], although only one report that we are aware of has documented vitamin D supplement use in Australians. In that report, 5% of Australian adults reported taking vitamin D supplements and 18% reported taking multivitamin supplements in 2011-2012 [1]. As expected, those who took vitamin D supplements had lower levels of vitamin D deficiency than those who did not take supplements (7% compared with 23%).

The aim of this study was to investigate the patterns of vitamin D supplementation in Australians between 2000 and 2010.

## 2. Methods

IMS Health is a leading provider of information to the health care industry. Data representing unit sales (as individual bottles) and dollar value in Australian dollars (Aus$1 ≈ US0.93) from manufacturers and wholesalers to pharmacies and hospitals for all vitamin D containing supplements was requested directly from IMS. IMS captures approximately 95% of total pharmacy sales. A panel of pharmacies is used to supplement any sales that are not captured or projected by imputation to give a national view to account for 100% of the market.

Data was obtained in Excel (Microsoft) for all manufacturers, including private label manufacturers and supplement brand name for each month between January 2000 and December 2010 for the following categories of vitamin D containing supplements: vitamin D plain, 100% vitamin D; vitamins A and D combination, containing some vitamin D; and calcium and vitamin D. Multivitamin supplements were not included as these generally were less potent than vitamin D or vitamin D and calcium supplements in Australia (personal observation).

In Australia, seasons are defined as follows: summer: December–February; autumn: March–May; winter: June–August; and spring: September–November. Descriptive analyses were used to describe the data in absolute values. The effect of season on vitamin D sales was investigated by ANOVA with Bonferroni* post hoc* analysis. A *P* value of 0.05 was used to determine statistical significance. Descriptive analyses and ANOVA were conducted in SPSS version 19.0 (IBM SPSS, USA, version 19.0).

## 3. Results

Eighty-nine manufacturers produced 195 different vitamin D containing products, with 26 (15.9%) of which containing vitamin D alone, 34 (17.4%) containing a combination of vitamins A and D, and 135 (69.2%) predominately containing calcium with or without vitamin D.

In terms of units sold, the calcium with vitamin D supplement market comprised the largest proportion, which was then followed by plain vitamin D supplements (32.4 million and 24.6 million units in 2010, resp.) (Table 1). However, when sales according to dollar value were compared for each product type, plain vitamin D supplements comprised the largest value, followed by calcium units (A$54.4 million and A$34.9 million units in 2010, resp.).

There was a consistent increase in wholesale vitamin D supplement sales from 2000 to 2010. During this period, the volume of vitamin D containing supplements sold almost tripled, increasing from 2.1 million to 6.10 million units, representing a value of A$34.1 to A$94.0 million in wholesale sales (Table 1).

**Figure 1:**
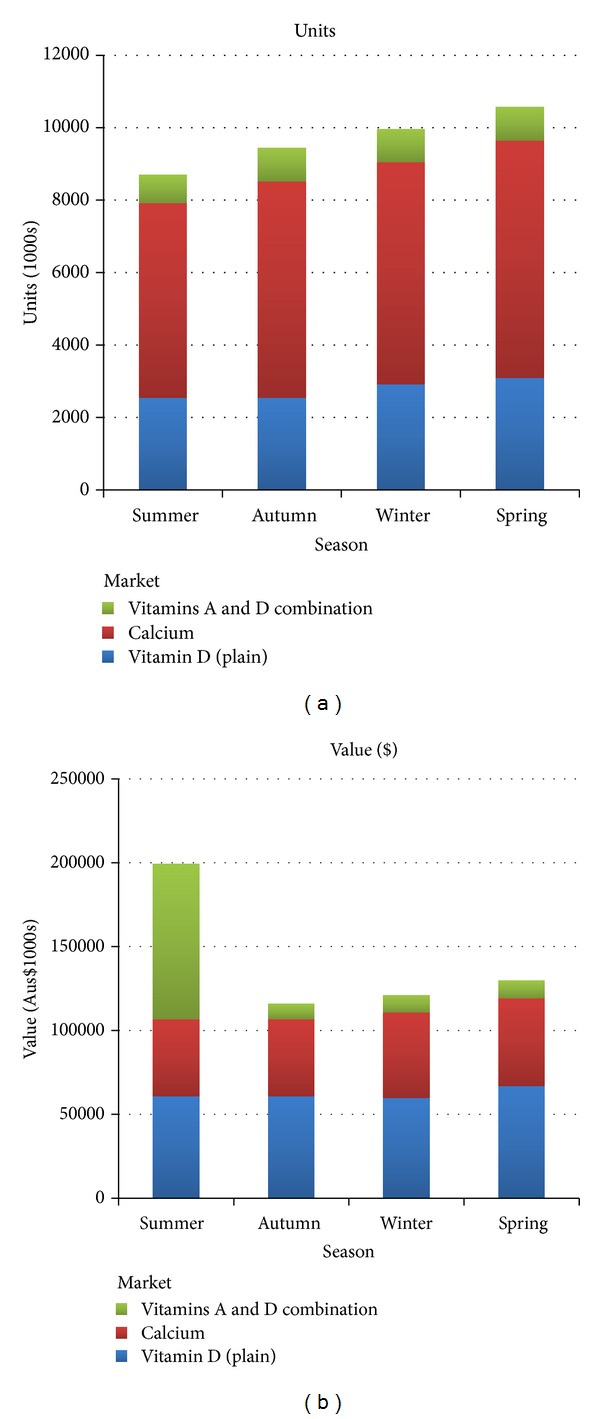
Total vitamin D supplement sales in thousands of units (units represent individual package as purchased from 2000 to 2010, source: IMS Health, Australia, 2012) and thousands of dollars ($Aus) from 2000 to 2010 according to season and product type.

As expected, a nonsignificant trend for increased vitamin D supplement sales from summer through spring was observed (Figure 1); however, the difference in sales did not reach statistical significance for units or value (data not shown).

## 4. Discussion

There has been an incremental increase in the use of vitamin D supplements as evident by the rise in vitamin D containing supplement sales. In 2010, more than A$94 million wholesale of vitamin D containing supplements was sold, of which over half contained only vitamin D. Based on a retail mark-up from anywhere between 50% and 75%, this equates to A$140–$165 million spent by consumers. Similar findings have been shown internationally. In an analysis of the NHANES III study, Gahche showed that the use of dietary supplements containing vitamin D increased from 29.7% of individuals in 1988–1994 to as high as 56.3% in women aged 60 and over in 2003–2006, whereas, in individuals aged 20–39, the rate remained around 22–26% for males and 30–34% for females [15].

The vitamin D content of supplements sold in Australia varies widely, with vitamin D only supplements containing the highest content of vitamin D per supplement (1000–2000 IU 25(OH)D per capsule). The content of vitamin D in vitamins A and D combination supplements also varied between approximately 100 and 400 IU. Calcium products contain a mix of supplements containing calcium alone or calcium plus vitamin D. If present, the vitamin D content in calcium products also ranges between approximately 40 and 1000 IU vitamin D. Our data do not include multivitamin supplements, which also provide vitamin D, and, as such, underrepresent vitamin D containing supplement use in Australians. However, multivitamin supplements generally contained lesser amounts of vitamin D than vitamin D only supplements (personal observation) and were, therefore, not included in this dataset. Individuals who take supplements have been shown to lead healthier lifestyles and consume better quality diets [16]. For instance, supplement users have been shown to have better dietary patterns, exercise regularly, maintain a healthy body weight, avoid tobacco products [16], and, therefore, obtain additional vitamin D from being out in the sun and eating foods containing vitamin D compared to those who do not use supplements.

Although a higher proportion of units of calcium supplements were sold (32.4 million units in 2010), vitamin D supplements compromised the highest proportion in regard to dollar sales (A$53.4 million). This is likely to be explained by the higher cost of vitamin D supplements relative to calcium supplements.

As previous reports have shown, the highest prevalence of vitamin D deficiency occurs in winter whereby 36% of Australians have been shown to be vitamin D deficient [1]. It would be expected that, following a diagnosis of vitamin D deficiency, individuals are more likely to purchase vitamin D supplement. A seasonal trend was observed where supplement sales increased linearly from summer to spring (Figure 1); however, the difference did not reach statistical significance (*P* > 0.05) for all supplements combined or by each product type. A drop in sales from summer would be expected on the basis that many Australians have access to adequate sunlight in the summer months. These figures represent absolute wholesale sales in units and Australian dollars to pharmacies and hospitals and not direct dates of purchase by individuals which may occur several weeks afterwards; thus, a trend may have been more evident if we had been able to investigate the lag in retail sales to consumers.

It is concerning that large amounts are spent on vitamin D supplements in Australia. Sunlight and food sources are alternative sources of vitamin D. Wide scale vitamin D testing of the Australian population in order to determine the need for supplementation would be expensive, as would universal vitamin D supplementation; therefore, neither of these is viable option. A simple tool to calculate the risk of vitamin D deficiency, which could then be followed up by a blood test if indicated, would potentially save the healthcare system a sizable amount of money and avoid potentially unnecessary testing and supplementation.

There has been an enormous increase in vitamin D awareness, testing, and supplementation in Australia since 2000. Supplementation may be the most feasible option for many individuals to reverse the high prevalence of vitamin D deficiency reported in Australians. However, it is possible that some individuals are unnecessarily taking supplements or, conversely, the dose of vitamin D in their supplement is inadequate and unlikely to assist individuals in achieving adequate vitamin D status.

## 5. Conclusion

Australians are spending enormous amounts of money on vitamin D supplements, a proportion of which may potentially be unnecessary. Further information is needed regarding the reasons individuals are taking supplements and the effect of such supplementation on serum vitamin D levels. Additionally, it would be useful to include multivitamin supplements in our analysis. Vitamin D supplementation guidelines are clearly warranted in Australia in effort to prevent unnecessary use of vitamin D containing supplements; and, conversely, for those who require vitamin D supplements to correct vitamin D deficiency, these guidelines would provide details on the correct dose required to lead to vitamin D repletion.

## Figures and Tables

**Table 1:** Annual vitamin D containing supplement sales in thousands of units^1^ and thousands of dollars according to year and product type.

Year	Units by product type (1000s)				Value by product type (A$1000)			
	Vitamin D (plain)	Vitamins A and D combination	Calcium plus vitamin D	Total units	Vitamin D (plain)	Vitamins A and D combination	Calcium plus vitamin D	Total value
2000	5.0	2.1	13.4	20.5	233.9	14.1	93.3	341.2
2001	5.0	1.9	14.7	21.6	216.3	12.1	104.0	332.4
2002	4.8	1.8	16.6	23.2	170.7	11.0	118.6	300.4
2003	5.1	3.1	18.3	26.5	144.2	30.2	132.8	307.2
2004	6.3	3.7	18.9	28.9	140.7	45.3	141.8	327.9
2005	6.9	3.5	20.9	31.4	141.6	44.7	162.0	348.2
2006	8.1	4.1	20.2	32.4	148.2	58.6	166.5	373.3
2007	10.6	3.7	24.4	38.8	177.3	51.8	210.2	439.3
2008	15.2	3.3	28.8	47.3	270.2	41.4	263.5	575.1
2009	19.6	3.8	31.9	55.3	408.3	48.1	316.5	772.9
2010	24.6	4.0	32.4	61.0	534.1	57.5	348.7	940.3

^1^Units represent individual items as purchased; source: IMS Health, Australia, 2013.
